# The role of social relationship in HIV healing and its implications in HIV cure in China

**DOI:** 10.1080/21642850.2015.1040405

**Published:** 2015-04-30

**Authors:** Shan Qiao, Jing-Bao Nie, Joseph Tucker, Stuart Rennie, Xiao-Ming Li

**Affiliations:** a Pediatric Prevention Research Center, School of Medicine, Wayne State University, Detroit, MI 48201, USA; b Bioethics Centre, University of Otago, Dunedin 9054, New Zealand; c Institute for Global Health and Infectious Diseases, University of North Carolina at Chapel Hill, Chapel Hill, NC 27599, USA; d Department of Social Medicine, University of North Carolina at Chapel Hill, Chapel Hill, NC 27599, USA

**Keywords:** HIV cure, healing, social relationship, China

## Abstract

HIV is both a biomedical disease and a social phenomenon that is constructed in particular cultural contexts. A successful and humane HIV cure requires not only the science of eradicating pathogens, but also the art of healing to restore harmony between mind and body. Healing in the context of HIV cure will be both personal and interpersonal, biological and social, and will involve rebuilding connections between HIV patients and their social environment. Social conceptions of healing have been highlighted in many regions with rich non-biomedical healing traditions, including China. Based on an adapted theoretical model on social relationships and health, we address the essential role of social relations for HIV healing in Chinese cultural context, and propose several recommendations for reforming practices and policies regarding HIV healing. In general, family is still a core social unit in HIV patients’ medical journey from diagnosis to treatment. A positive patient–physician relationship based on mutual respect and trust also has critical impact on patients’ physical and mental health. Physicians may become a key or the main source of social support in circumstances when families are not actively engaged in healing. Reconnecting HIV patients with their communities should be a necessary component of HIV cure, as this will help patients engage more fully in the HIV healing process. We call for a family-centered approach in HIV healing intervention to strengthen patient–family ties; a series of policies to build up and sustain positive patient–physician ties; and multi-level strategies to empower patients and rebuild their bonds to community and larger society. We also call for more empirical research on how non-biomedical healing approaches in various cultural settings could (directly or indirectly) inform HIV cure research.

## Introduction

Dashan, a 37-year-old male patient from a small town in Guangxi, China, was diagnosed with HIV infection four years ago (The case comes from an unpublished study based on qualitative data collected in 2012 from Guangxi, China. We use pseudonym here.) He went to a hospital for continuing cough and fever, and then received antiretoviral therapy (ART) treatment immediately after the HIV diagnosis. He talked about his experience in a calm and optimistic tone:

When I first came to the hospital I had no idea what kind of disease I had. My family, my brothers did not tell me even I asked them about it. They were afraid I might commit suicide when I knew the truth. They told me the fact later when I got much better. But I figured out that I had HIV by talking with other patients in the same ward. I fell in despair and preferred dying at that time. My families were always watching me during my hospitalization in fear of my committing it [suicide].

I am very close to my father, and I would like to tell him everything including the problems I have. Thus he was the first person I talked with about my disease. I always trust him and accept what he says. When I knew the diagnosis I did not want to live any more. It was him that encouraged me to move on. My brothers paid all the expenses of my hospitalization, and they always give me money. My sister is living near me. She said, “no matter what happens, I am just a phone call away. We don’t care how much money we spend, as long as you get better. We are a family.” When I need to visit a doctor for a cold or fever, she always gives me a ride to the hospital.

I can’t stay here today without my family. And I especially appreciated my wife, for her not leaving me. She treats me as same as before. Now my daughter is 6 years old. We plan to have another child. My wife loves kids. We have counseled with doctors in Nanning, and they told us it possible to have a healthy baby in our situation.

Dr. Zheng in the hospital was a very nice man. He told me how to apply for subsistence allowances from the government, and introduced me to the Red Ribbon Program (a local support group in Guangxi). I felt better when I joined the support group of HIV patients. I didn’t fear of that disease so much when I saw other patients were having a positive life. Now my CD4 is good, and I am confident in living a healthy life for my family.

Dashan’s story provides us a glimpse into a part of illness experiences among HIV patients in China, revealing that different social relations have played a profound role in their healing process. The concept of healing has three dimensions: wholeness, narrative, and spirituality (Egnew, 2005). Healing involves achieving wholeness as a person in physical, emotional, and social aspects. Healing occurs in the life stories of the person experiencing illness within social connections with others. Healing also means acquiring a harmony between the body, the mind, and the spirit (Egnew, 2005). Clearly, if curing HIV is conceived in terms of healing, HIV is a social phenomenon, constructed in particular contexts, and not just a biomedical disease. Given the complicated meanings of HIV to patients, their family, and society, the HIV healing process will involve diagnosis, disclosure, access to treatment, medication adherence, self-care and disease management, and reconnections between HIV patients and their social environment. In the era when HIV has transformed from a fatal disease into a treatable, chronic, and now potentially curable disease, there is increasing agreement that curing HIV will involve far more than eliminating the HIV virus from patients’ body; it will also be a process of healing embedded in social relationships and cultural meanings (Tucker & Rennie, 2014).

The role of social relationship in the general healing process has been highlighted by studies from multiple disciplines. Ethnographies and anthropological studies have demonstrated how social relationship was used as a main component in many non-biomedical traditional healing practices. For example, Ojibwa living in Canada and Ndembu in West Africa utilize public confessions of patients about their “wrong” deed toward others in the community as a mechanism for transforming ill-feeling into well-wishing, for restructuring relationships to restore social harmony, and for reintegrating the sick person into the social group (Hallowell, 1963; Turner, 1967). Empirical studies have suggested the positive role of social relationship in healing in terms of improving patient care, increasing compliance with medical regimens, and promoting decreased length of hospitalization (Holt-Lunstad, Smith, & Layton, 2010). By comparison and reflection on diverse healing systems, scholars have called for bringing back social relations between patients and others (their families, physicians) within the biomedical practices of diagnosis and treatment (Kleinman, 1980; Miller & Crabtree, 2005).

In the area of HIV studies, there is an increasing reflection and critique on distinction between biomedical and social dimensions of HIV, and calling for the response to HIV should engage with the everyday lives of people and be integrated into their social relations and social practices (Kippax and Stephenson, 2012; Auerbach, Parkhurst, & Caceres, 2011). A growing number of studies have explored the influence of various social relations in the HIV healing process, for example, the role of social relations in self-management among women living with HIV (Webel & Higgins, 2012), the effect of physician–patient relation in appointment adherence and HIV viral suppression (Flickinger, Saha, Moore, & Beach, 2013), the association between quality of relationship with partner and ART adherence (Johnson et al., 2012).

However, there is a dearth of theoretical reviews or conceptual frameworks on how social relationship may affect the HIV healing process. One of potential theoretical sources could be the theories on social relationship and health including the stress buffering and main effects models (Cohen, Gottlieb, & Underwood, 2000). The first model posits that the aid from social relationships moderates or buffers the negative effects of stressors on health (Cohen, Gottlieb, & Underwood, 2001); the second one proposes that social relationships may directly or indirectly encourage healthy behaviors and being part of a social network provides self-esteem and purpose to life (Cohen, 2004). Based on these existing theories, we hypothesize that social relationships (e.g. patient–family relations, patient–physician relations, and patient–society relations) may influence the HIV healing process through three different pathways: social support, social integration, and social norms.

Social support refers to perceived or actual psychological and material resources provided by a social relationship (Cohen, 2004), including instrumental, informational, and emotional support (House & Kahn, 1985). Social support can reduce impacts of stressful experiences, facilitate medication adherence among HIV patients, improve their quality of life, and assist them to access more resources needed to resolve financial problems (Rao et al., 2012; Tsai et al., 2012; Wohl et al., 2011).

Social integration is defined as participation in a broad range of social relationships (Brissette, Cohen, & Seeman, 2000). High level of social integration implies active engagement in various social relations (social network) and social activities, and a sense of belonging and identification with one’s social roles (Brissette et al., 2000). Social integration can also promote identity and self-worth as well as provide motivation and social pressure to care for oneself (Cohen, 2004). Social integration has been shown to impact physical and psychological health, HIV self-management, and medication adherence among HIV patients (Campbell et al., 2013).

Social norms are the (explicit or implicit) rules that a group uses to distinguish appropriate and inappropriate values, beliefs, attitudes, and behaviors. Failure to follow social norms can, in some contexts, result in severe punishments (Bicchieri & Muldoon, 2014). Social norms may affect the HIV healing process in a complex way. For example, gender norms and gender inequality in many societies make females vulnerable in HIV care and treatment. Women may fear of disclosing their HIV-positive status to their spouses due to potential domestic violence after disclosure (WHO, 2004). It may be more difficult for them to access HIV care service (Aziz & Smith, 2012) and maintain ART treatment (Wang et al., 2011). Community norms toward HIV/AIDS may influence medical seeking among HIV patients through their perceived stigma from the community. One study conducted in New England communities in the USA indicated that HIV patients perceived less stigmatized in communities where residents reported high internal motivation to control HIV-related prejudice and stigma (Miller, Grover, Bunn, & Solomon, 2011).

In addition to the three proposed pathways, cultural context should also be a component of the framework on social relationship and HIV healing. Social relationships, healing practices, and the meaning of healing are embedded in particular social and cultural settings (Kleinman, 1980). HIV patients in diverse social environments may respond and interpret their illness in different ways and experience various medical journeys. China has a long history of non-biomedical traditional healing practices and complicated healing knowledge systems deeply influenced by not only Confucianism but also by Daoism and imported Buddhism (Nie, 2009). These medical beliefs and practices have constructed the Chinese cultural context of healing (Nie, 2009). In this paper, we will briefly review the Chinese cultural context of healing, explore how social relationship may influence HIV healing in Chinese culture, and discuss several implications for reforming practices and policies regarding HIV healing.

## The Chinese sociocultural context for healing

Chinese culture has been widely characterized as communitarian, collectivistic, and familistic with high emphasis on social relationships and interpersonal networks (*guanxi*). Typical social relationships under Confucianism embody the following characteristics: first, family relationships constitute the core of social life. The interests and needs of the whole family are highlighted and regarded as prioritized over that of any individual members (Cong, 2004). Second, social relationships are reciprocally obligatory in the long term. Calculating immediate personal profits is antithetical to the principle of mutual faithfulness and trust. Third, superior groups have authority and power over inferior groups, reinforcing the basic hierarchy of power in traditional Chinese society. Fourth, individuals need to maximize their value of life through keeping harmony with various social relations.

The healing practice in China has been deeply influenced by its culture that highly values social relationship and social harmony. Corresponding to the features of social relationships, traditional Chinese healing practice dictates that (1) family plays a crucial role in deciding strategy for best interests of the family and the individual patient. The family is expected to provide support during the patient’s illness, and has responsibility and authority to communicate on the patient’s behalf (Kim, 2005; Tsai, 2005); (2) physician and patient should develop long-term mutual trust with each other. As a famous physician in Chinese history asserted,

If patient places his trust in a physician, he should develop this trust over a long period of time, and not on short notice…Faced with those, however, in whom I place my trust on short notice [in the case of a crisis], there arise doubts and confusion. (He Qibin [Ho Ch’i-pin], Unschuld, 1979, P111)

(3) A physician’s authority is highly respected. Patients expect that their physicians will act in their best interest. They are socialized to accept information and follow instructions from physicians without asking many questions or expressing concerns (Chen, Starks, et al., 2007). (4) Being in harmony with one’s social environment is helpful in promoting health and healing illness (Chen, 2001).

These principles of traditional healing practice suggest that we need to pay close attention to three critical social relationships during HIV healing: patient–family relations, patient–physician relations, and patient–society relations. However, care should be taken not to subscribe to some pervasive stereotypes that view Chinese culture as monolithic and static entity. Historically, China’s other major philosophical and religious traditions, Daoism and imported Buddhism, have also profoundly shaped healing practice in China (Needham, 1956; Unschuld, 1985). As an opposite to Confucianism, both Daoism and Buddhism underscore personal freedom. The sweeping wave of modernity and socialism contributed to the radical changes of the traditional social structure and functions of families and communities. Therefore, it is thus critical to acknowledge the great plurality and dynamics of Chinese cultures and society including the healing systems and practices (Nie, 2009, 2011). For health care and HIV healing, this means that we should fully consider the specific needs of different social groups as well as the unique social setting of each and every individual patient.

## The essential role of social relations for HIV healing in China

### Family: restoring an elementary social unit in crisis

Family is an elementary social unit for anyone facing the world’s challenges and stressful events. Existing studies suggest that the patient–family relationship shapes the healing process through mechanisms of social support, social integration, and social norms. Family is supposed to be a main source of social support that HIV patients may rely on. However, the process and consequences of support seeking may vary significantly depending on the status of people involved in a certain relationship. Some families accept HIV patients and provide all types of support, deepening bonds of family members, finding new strength through facing challenges together (Chen et al., 2011). Some families reject and even expel patients from the family (Zhou, 2007). Some patients choose not to seek support or even deliberately distance themselves from their families in order to protect their loved ones from potential psychological burden (Qiao, Li, & Stanton, 2014).

Relations with families can influence patients’ integration into society in terms of a behavioral component (social interaction) and a cognitive component (identification of one’s social roles). For example, isolation from families undermines the social integration by reducing patients’ engagement in various social relationships and social activities. Perceived or actual failure to fulfill obligations toward families results in self-blame, sense of alienation, and decreased feelings of control. These negative psychological outcomes constantly impede patients’ self-esteem in their daily life and threaten their self-identity in the larger society. In one extreme case, an HIV-positive woman worried about her ability to bear healthy sons to carry on the family name and insisted on a divorce. In another case, an HIV-positive wife was willing to tolerate infidelity of her husband because she did not view herself as a “complete” wife due to her illness. She did not consider that she would be entitled to faithfulness from her partner (Chen et al., 2011). Some patients are bothered by their inability of fulfilling filial duties to parents (Zhou, 2007). On the other hand, the patients’ desire to fulfill their social responsibilities may stimulate a strong motivation to protect their health and live a long life. For many patients with children, their affections and obligation toward children are important reasons for their adherence to HIV treatment.

Social norms on disease disclosure in China reveal tensions between family authority rooted in Confucian collectivism and increasing ideal of individual autonomy, which contributes to various practices of HIV disclosure and dilemma encountered by the physicians in hospitals who may not be HIV testing or counseling experts. Some physicians and families of patients believe that hiding an HIV diagnosis will protect patients from psychological problems (as Dashan’s story illustrates). In HIV healing practice, many physicians treat HIV patients similar to cancer patients. They assess the patients’ conditions and their family situations to make a decision about how to inform them about the HIV diagnosis (Li, Lin, et al., 2009). The physicians usually chose a family head (parents) or other core family member (spouse) for notification. By doing so, they also hope/assume patients’ family to be involved in treatment and care as early as possible (Li, Lin, et al., 2009). However, this approach involves the violation of patient confidentiality and autonomy by the physician. Patients may become furious with the physicians for unintended consequences of HIV disclosure (such as family conflicts).

Some physicians believe that patients have the right to know their own health conditions and should have decision-making power related to disclosure of their HIV status to others. They usually first inform patients themselves and encourage them to inform their family and especially their sexual partners for secondary prevention (Chen et al., 2011). However, physicians often have difficulties in persuading their patients to bring their sexual partners to be tested for HIV. The physicians are not able to guarantee their patients would like to disclose HIV status to their partners and/or they can protect their partners by using condoms during sexual intercourse. Many physicians feel that they are in a moral dilemma between protecting patients’ confidentiality and preventing secondary transmission. They also struggle with conflicts between the demands of policy and regulations regarding HIV testing and the realities of notifying patients about their HIV status.

### Patient–physician relationships: trust and medical professionals’ duty to care

The patient–physician relationship is a key component in the healing process. Existing studies suggest that it can either positively or negatively influence HIV healing practice through social norms and social support. In a long medical history of China, a positive patient–physician relation has been valued and emphasized. Ideally, the patients respect the physicians’ authority and thus implicitly trust them in their information and decision on treatment; for their part, physicians are expected to act in patients’ best interest and treat them with compassion:

[A Great Physician] should not give way to wishes and desires, but has to develop first of all a marked attitude of compassion…He should look upon those who have come to grief as if he himself had been struck, and he should sympathize with them deep in his mind. (Sun Simiao [Sun Szu-miao], Unschuld, 1979, P30)

In HIV healing practice, most physicians and patients believe in the socially regulated ideal of the patient–physician relationships. Physicians’ perceived responsibilities to care for patients ensure that they maximally use their professional abilities to treat patients. The trust of patients in their physician’s expertise and authority contributes to their positive and effective interactions with the physicians during the healing process in terms of sharing personal health information with physicians, engaging decision-making, and conducting self-care activities (Chen et al., 2013).

For patients rejected and isolated by their families, the social support from their physicians is critical to maintain both physical and mental health (Zhou, 2009). In addition to medical care, the support from physicians may include emotional support, informational support on HIV treatment, and financial support (by accessing resources to ease financial burdens) (Chen, Starks, et al., 2007). Social support from physicians can positively impact patients’ medical adherence, life quality, and even their relationships with families, for example, by giving explanations and advice to patients’ family members about HIV treatment or secondary prevention (Chen, Starks, et al., 2007; Wang et al., 2008; Zhou, 2009).

### Community and the larger society: healing HIV through rebuilding connections

Because health is tied to a sense of connection to social environment (Loxterkamp, 2013), healing a patient means rebuilding the connection between the patient and his/her community which has been broken by illness. In the practice of HIV healing, social integration and social norms may play an important role during the process of reconnecting HIV patients with their community.

Social integration can facilitate such reconnection through engaging HIV patients in social activities, improving their self-worth and strengthening the sense of belonging. Based on the empirical studies, effective approaches of social integration among HIV patients include social support groups and community-based organizations. Social support groups and peer group/counseling have proved to be effective in decreasing psychological distress, improving medication adherence, and enhancing quality of life among HIV patients in China (Molassiotis et al., 2002; Simoni et al., 2011). Community-based organizations can also strengthen social integration through connecting HIV patients and social resources for HIV prevention, treatment, and care (Zachariah et al., 2009). One pilot study in Jiangsu has suggested that it is feasible and cost-effective to shift essential HIV-related service from government systems to local community-based organizations with respect to improving the coverage of HIV testing among men who have sex with men, CD4 tests, and ART therapy among HIV patients (Yan et al., 2014).

Social norms on HIV patients may compromise the reconnection between HIV patients and their communities by impeding the efforts from both the community and the patients. Despite the development of medical treatment that has transformed HIV into a chronic and manageable disease, social norms on HIV patients are still severely affected by stereotypes and prejudice (Chen, Choe, Chen, & Zhang, 2007; Li, Wang, Williams, & He, 2009). Because some transmission routes of HIV were closely related to “deviance” from socially accepted standards of normality (e.g. drug use, commercial sex), acquisition of HIV is easily viewed as being socially or morally wrong. Some populations with most risks of being affected by HIV, including sex workers, drug users, and men who have sex with men, were already highly marginalized by their communities independently of HIV (Zhou, 2007). The evident and persistent HIV-related prejudice and stigma have been a barrier for community members to understand and accept HIV patients, or involve them in daily social activities (Katz et al., 2013).

Social norms toward HIV patients may also shake HIV patients’ confidence in reconnecting with their community by encroaching their self-worth and self-identity. The process of a person losing his or her sense of self is determined by daily interactions with the world outside of themselves (Alonzo & Reynolds, 1995). Stigma makes them feel shamed and guilty for their individual “deviance” from their social roles and potential violation and harm to the social relationship they cherish (Goffman, 1963). Concealing HIV infection, being anxious of unexpected breach, and “living a double life” may psychologically overwhelm HIV patients and undermine their self-esteem (Zhou, 2007). They may lose their feelings of belonging to some social relations and lose confidence in maintaining and developing connections with their communities (Zhou, 2007).

It is notable that social norms toward HIV patients may be various due to their HIV transmission routes. A large number of HIV patients in China were infected through blood transfusion, blood products, or commercial blood donation. They were viewed as “innocent” rather than “culpable”. The moral meanings and social stereotypes about infection modes are manifested among HIV patients themselves, which greatly impact their HIV healing process (Chan, Yang, Zhang, & Reidpath, 2007; Zhou, 2007).

## Recommendations for reforming practices and policies

Family relationships are believed to have greater emotional intensity than most other social relationships and thus provide significant support and care for HIV patients (Rochat, Mkwanazi, & Bland, 2013). There is a growing acknowledgment of the key role of family in HIV care and treatment (Wouters, 2012). We recommend family-centered approach interventions in the HIV healing process, with aims to engage the family members of HIV patients in the HIV disclosure, counseling, and medical care. The first strategy of these interventions is to strengthen the family relationship. They could encourage family engagement and interaction by involving HIV patients and their family members in same activities (e.g. games, sports, other entertainment activities, group meetings, etc.). The interventions could also focus on improving skills of emotional management and effective communication for HIV patients and their families. These skills of interpersonal communication are necessary for a positive discussion of disease and enhance trust and understanding between family members.

The second strategy of the family-centered interventions is to assist the family affected by HIV adjust to the changes brought by HIV and reduce their stress and social isolation. Based on the assessment of family relationship and discussion with HIV patients, the health care workers can consider inviting family members of HIV patients to join in the HIV counseling so they can learn more knowledge of HIV prevention and treatment, reduce the serotypes and misconceptions on HIV, and provide potential social support. Family members can also be encouraged to participate in daily disease management and positively affect the medication adherence of HIV patients. Empirical studies in sub-Saharan Africa suggested that engaging family members as treatment partners for HIV patients was associated with improved medication adherence (O’Laughlin, Wyatt, Kaaya, Bangsberg, & Ware, 2012). A number of approaches to providing couples-based support for adherence and HIV prevention have also been developed and examined (El-Bassel et al., 2010; Wrubel, Stumbo, & Johnson, 2010).

The third potential strategy for family-centered intervention is to address the needs of family members, particularly the care providers of HIV patients within their family. In many families affected by HIV, grandparents or young children have to undertake the tasks of providing daily care for HIV patients. They may have to face multiple challenges and social and health problems (Moradi, Miraghaei, Parhon, Jabbari, & Jobson, 2013). Psychological counseling and social support will be helpful for this vulnerable group to prevent them from sacrificing normal family life and losing self-efficacy in coping with HIV (Rutakumawa, Zalwango, Richards, & Seeley, 2015; Lane, Cluver, & Operario, 2014).

To facilitate a long-term, positive patient–physician relationship, we propose three strategies to improve the path toward HIV healing. First, it is necessary to refine existing regulation and policies about HIV diagnosis notification and HIV disclosure to patients’ sexual partners or other family members. The dilemmas encountered by physicians may increase the likelihood of physicians violating their patients’ confidentiality and trust. In addition, physicians need intensive training on how to communicate HIV testing results to patients, and how to assist patients to disclose HIV status to their sexual partners and family members. Appropriate disclosure is the first and key step to build up trust between patients and physicians and also an important issue patients need to face for seeking support from patients’ families. This approach will help build trust between patients and physicians that will be critical for HIV healing.

Second, we call for interventions or services to meet psychological needs among physicians in the frontline of HIV healing. It is hard to expect a physician who endures internal shame or perceived stigma due to working with HIV patients to provide his/her patients with emotional support. Institutional support (e.g. specialized HIV-related training, sufficient supply of sterile materials, and universal health insurance coverage for occupational exposure) will promote positive psychological states among HIV physicians, maintain a stable workforce, and improve the quality of care for HIV patients (Li et al., 2007). In addition, the psychological well-being of physicians is inevitably affected by negative emotions and experiences triggered by social norms and attitudes toward HIV/AIDS. Reducing stigma against HIV patients in the general society and building an environment in which HIV physicians feel protected and comfortable to provide healing should also be important steps to improve psychological well-being of HIV physicians.

Third, we call for decentralized care models for HIV to address the challenges of limited technical expertise and human resources in HIV healing. We propose a change from the current centralized facility-based model (e.g. government public health agencies) to a community-based model in which community health workers (CHWs) assume the central responsibility for the promotion of treatment and care (Xu, Zhu, Gao, Liu, & Du, 2012). Living in the same community, CHWs are likely to develop long-term mutual trust relationship with HIV patients. In addition, CHWs have a great chance to be familiar with patients’ family circumstances and thus play a positive liaison role between patients and their families in disclosure and treatment practice. By integrating case management (e.g. regular follow-up) for HIV patients into regular family primary health care (e.g. child vaccination), CHWs may reduce the cost of providing HIV-related care, help avoid unexpectedly breaching the HIV status of patients, and normalize HIV treatment and care in local communities (Zhang, Miege, & Zhang, 2011).

To rebuild the connections between HIV patients and their communities or even larger society, we highlight the important role of social integration in HIV prevention, treatment, and care. First, the government should encourage the development of community-based organizations among HIV patients and engage them in HIV service delivery. Community-based organizations can be a platform for HIV patients to participate in social activities and build up new social relationships with their peers and other members in the society (such as volunteers, paraprofessionals, etc.).

Second, we call for more attention to the strengthening of self-worth and self-care capacity among HIV patients. In the age of HIV cure, HIV patients will assume responsibility for their treatment and develop the capacity for self-care similar to patients with diabetes and asthma (Adams, Pill, & Jones, 1997; Aujoulat, Marcolongo, Bonadiman, & Deccache, 2008). The self-management of chronic disease requires patients to be fully active participants in the healing process. The existing literature reveals that patients are often treated as inferior in their relations with physicians in many circumstances. To empower HIV patients, physicians should be trained to respect the dignity of patients, protect their privacy and confidentiality, encourage patients to ask questions and express their concerns, and to apply effective communication skills (e.g. active listening, mindfulness, and emotional intelligence) in clinical encounters.

Third, we need considerable investment of time and resources to reduce and eradicate HIV-related stigma and discrimination which is one of the main barriers for the reconnection between HIV patients and their community during HIV healing. The intersection of HIV-related stigma and social disparity should be considered in the design of interventions and the allocation of resources so the efforts to reduce stigma and discrimination associated with HIV can benefit the most vulnerable and marginalized populations. In addition, the stigma-reduction interventions and activities should involve HIV patients as stakeholders so they can be empowered through fighting for themselves.

## Conclusion and limitations

Illness experience is embedded in a variety of social relationships. Lifelong effective ART can suppress viral replication to very low levels, or even eradicate virus. However, technology itself is unable to restore harmony between mind and body for HIV patients (self-identity) or fix broken bonds between patients and their social environment (social relationships). Our brief review of HIV healing has demonstrated that the features of social relationship related to health care in China are influenced by Confucianism and traditional Chinese medical practice.

When exploring the role of social relationships in HIV healing in China, one must adequately acknowledge the complexity and dynamics of Chinese healing systems. China has been experiencing a huge social and cultural transformation since the late nineteenth century, in which Confucianism and traditional medical practices have been doubted, criticized, rediscovered, and reinterpreted when they encountered other beliefs, ideologies, and techniques. Current HIV healing practices embrace a diverse group of healers, from shamans, village doctors (with limited medicine training), to physicians; as well as various techniques from religious rituals, herbs, acupuncture, to medicine and surgeries depending on patients’ residence, ethnicity, social and economic status and their medical knowledge and beliefs. HIV patients may utilize multiple healing strategies to deal with their disease. The different patterns of HIV healing experiences deserve further studies from a multi-disciplinary perspective.

In conclusion, social relationships have a profound impact on current HIV healing in China. HIV patients’ relations with family and physicians are main factors that reshape HIV disclosure, social support, and self-identity. For HIV patients, a resilient family and a positive mutually respectful relation with physicians are helpful to constructively understand this disease, to actively engage in self-care and self-management, and to improve quality of life. These positive relationships with others are also significant for them to rebuild self-esteem, reconstruct AIDS disclosure in daily life, facilitate their social networks transforming into anti-discrimination forces in their community, and gradually generate changes in the larger society.
